# Hemocyte-Mediated Phagocytosis in Crustaceans

**DOI:** 10.3389/fimmu.2020.00268

**Published:** 2020-03-03

**Authors:** Shan Liu, Shu-Cheng Zheng, Yan-Lian Li, Jun Li, Hai-Peng Liu

**Affiliations:** ^1^State Key Laboratory of Marine Environmental Science, State-Province Joint Engineering Laboratory of Marine Bioproducts and Technology, Xiamen University, Xiamen, China; ^2^Laboratory for Marine Biology and Biotechnology, Pilot National Laboratory for Marine Science and Technology, Qingdao, China; ^3^Department of Life Science and Engineering, Jining University, Qufu, China; ^4^School of Science and Medicine, Lake Superior State University, Sault Ste. Marie, MI, United States

**Keywords:** phagocytosis, hemocyte, innate immunity, white spot syndrome virus, crustacean

## Abstract

Phagocytosis is an ancient, highly conserved process in all multicellular organisms, through which the host can protect itself against invading microorganisms and environmental particles, as well as remove self-apoptotic cells/cell debris to maintain tissue homeostasis. In crustacean, phagocytosis by hemocyte has also been well-recognized as a crucial defense mechanism for the host against infectious agents such as bacteria and viruses. In this review, we summarized the current knowledge of hemocyte-mediated phagocytosis, in particular focusing on the related receptors for recognition and internalization of pathogens as well as the downstream signal pathways and intracellular regulators involved in the process of hemocyte phagocytosis. We attempted to gain a deeper understanding of the phagocytic mechanism of different hemocytes and their contribution to the host defense immunity in crustaceans.

## Introduction

The fundamental theory of phagocytosis was first described by Élie Metchnikoff in 1882, which has been gradually established and well-understood over the past two centuries (1). Phagocytosis currently is described as an endocytic process that endogenous foreign particles or pathogens larger than 0.5 μm were first recognized by phagocyte surface receptor and then uptaken and engulfed into a plasma-membrane device, known as phagosome, following initiation of a signaling cascade to generate phagolysosome by fusion of phagosome with lysosomes. Finally, particles or pathogens within the phagolysosome will be degraded and cleared by the hydrolytic enzymes (2, 3). Phagocytosis has been considered as an essential defense mechanism of immune response to pathogens among eukaryotes, which are also implicated in diverse physiological processes, including development, apoptotic, tissue repair, and host defense (4). Owing to its importance and contributions to the innate and adaptive immune function in relation to human and animal health, phagocytosis still remains of great interests to many scientists.

Nowadays, aquaculture industry has become one of the most important resources for providing the people of the premium animal proteins, and employments over the world, especially in China and many Southeast Asian countries (5). However, the rapid expansion and development of aquaculture industries has been significantly inhibited due to infectious diseases, the key challenge imposed to sustainable aquaculture in China. Particularly, *Vibrio* bacteria and white spot syndrome virus (WSSV) have been recognized as the main threats among these pathogens in crustacean (6, 7). However, no effective strategies are available so far to control the outbreak of infectious diseases in crustacean aquaculture due to lack of knowledge about host–pathogen interaction, in particular the poor understanding of the host defensing immune function in crustaceans.

During the past few decades, hemocyte-mediated phagocytosis, as one of the most important innate cellular immune function, has also received great attention in crustacean, and a good progress in elucidating the involvement of hemocyte-mediated phagocytosis, as well as its protective roles and mechanisms, against bacterial and viral infections has been achieved. In this review, we summarized the recent progress about phagocytosis of pathogens by hemocyte in crustaceans, in particular focusing on the novel findings about related receptors for recognition and internalization of pathogens as well as the downstream signal pathways and intracellular regulators involved in the process of hemocyte phagocytosis. We attempted to gain a deeper understanding of the phagocytic mechanism of different hemocytes and their contribution to the host defense immunity in crustaceans, which will be beneficial for the establishment of potential effective strategies to control diseases caused by viruses and bacteria in crustacean industries.

## Biological Characteristics of Hemocyte

Phagocytes occur in many species, with extreme variations in abundance, evolving from the most primitive unicellular organisms, such as amoeba *Dictyostelium discoideum*, to higher multicellular vertebrates. Phagocytes in higher vertebrates, like mammalian species, have been highly specified into multifarious phagocytic cells, including neutrophils, eosinophils, monocytes/macrophages, dendritic cells, and osteoclasts, and termed as professional phagocytes, whereas others, so-called non-professional phagocytes, mainly include epithelial cells (8). However, regarding invertebrates, such as insects and crustaceans, phagocytes are kindly equivalent to hemocytes, which not only are involved in encapsulation, coagulation, and melanization but also exhibit phagocytic activities (9). Based on limited knowledge on crustacean hemocytes, three distinct types of hemocytes, named as hyaline cells, semigranular cells, and granular cells, have been classified and identified in most of crustaceans mainly based on cell size, nuclear/cytoplasmic (N/C) ratio, and the number of intracellular granules (10, 11). Hyaline cells have been characterized by their smallest cell size, having none or very few cytoplasmic granules, and the highest N/C ratios, while granular cells are the biggest cells with lowest N/C ratios and filled with larger cytoplasmic granules within hemocytes. As for semigranular cells, they are featured as their middle cell size between granular and hyaline cells, with smaller N/C ratios, and contain more cytoplasmic granules, but the number of granules is less than that of granular cells. Further molecular markers have also been proposed in a previous study for distinguishing different hemocytes of signal crayfish *Pacifastacus leniusculus*, and their results showed the potential application of superoxide dismutase (SOD) and two-domain Kazal proteinase inhibitor (KPI) to differentiate the granular and semigranular cells, respectively; however, more detailed molecular evidences are still required to further confirm their specificity in hemocyte classification (12).

Given its essential role in innate immune system, hemocyte homeostasis is of great importance for the health of crustaceans. Actually, the crustacean hematopoiesis has been well-investigated and described in several species since it was first reported in the early 1800s, such as shore crab *Carcinus maenas*, the lobster *Homarus americanus*, and signal crayfish *P*. *leniusculus* [an extensive review is made available by Lin and Söderhäll (13)]. For example, a study on signal crayfish *P*. *leniusculus* indicated that their hematopoietic tissue (Hpt) contain at least five different cell types corresponding to various developmental stages of granular and semigranular cells (11). Type 1 cells may be the precursor stem cells for the different cell lineages, and type 2 cells may be the precursor of granular and semigranular cells, both of which are the main cell types in Hpt. Types 3 and 4 may be the precursors of granular cells, whereas type 5 cells may lead to differentiating to semigranular cells (13). For the phagocytic capacity of different subpopulation of hemocytes in insects, previous studies demonstrated that plasmatocytes are the main phagocytic hemocytes in *Drosophila*, while granular cell and plasmatocyte are the main phagocytic hemocytes in Lepidoptera (14). Similarly, different subpopulation of hemocytes in diverse species or even in the same species of crustaceans also exhibited variable phagocytic activities. For instance, the higher phagocytic capacity has been observed in the hyaline cells rather than granular and semigranular cells of *C. maenas, Carcinus aestuarii* (15), and *Eriocheir sinensis* (16, 17), while main phagocytic capabilities of granular and semigranular cells were observed in *Macrobrachium rosenbergii, Penaeus monodon*, and *Cherax quadricarinatus* (17–20). Higher phagocytic activities were also demonstrated in the semigranular cells of signal crayfish *P*. *leniusculus*. In contrast, the phagocytic activity of semigranular cells was weaker than that of granular cells in *Scylla paramamosain* (15). However, it is noteworthy to mention that different subpopulations of hemocytes seem to exhibit specific preferences in phagocytosis of different bacteria or viruses. For instance, *Escherichia coli* was mainly ingested and cleared by semigranular and granular cells, whereas WSSV was mostly ingested by semigranular cells in red claw crayfish *C*. *quadricarinatus* (20). Although phagocytosis has been found in different subpopulation of hemocytes in distinct species, thus far, it is still very difficult to clearly classify the high amounts of evolutionary diversity of crustaceans. Meanwhile, the differentiating and developmental mechanisms of different subpopulations of hemocytes are also unclear. Therefore, more researches especially focused on fundamental theories still need to better characterize the characteristics of subpopulations of hemocytes and their corresponding phagocytosis in crustaceans.

## Receptor or Opsonin-Mediated Pathogen Recognition

In mammals, microorganisms are initially recognized by phagocyte receptors, including Fcγ receptor, complement receptor, fibronectin receptor (α5β1 integrin) and launch phagocytosis (3). Furthermore, the process of phagocytosis can be facilitated once pathogens were coated with opsonins (known as opsonization) because both opsonins and pathogen-associated molecular patterns (PAMPs) on the surface of pathogens are in turn easily recognized by phagocyte receptors. Phagocytosis mediated by hemocytes has been a great contribution to the defense in crustaceans against various pathogens, including *Vibrio parahaemolyticus, Vibrio harveyi, Staphylococcus aureus, Aeromonas hydrophila*, and WSSV. The receptors on the surface of hemocytes, such as lectins, scavenger receptors, immunoglobulin-related protein, and fibrinogen-related protein, have been reported in relation to phagocytosis for fighting pathogens (21–24). In the following, we summarized and discussed the recent advances on the involvement of related receptors or opsonin of hemocytes and their mediating recognition of pathogens in crustaceans.

### Lectins

Lectins are featured by carbohydrate recognition domain (CRD) that consists of ~110–130 amino acids and binds to carbohydrates. So far, many groups of lectins, including C-type, F-type, I-type, L-type, M-type, P-type, R-type, chitinase-like lectins, ficolins, calnexin, galectins, and intelectins, have been identified in crustaceans (21). It has been reported that lectins can act as opsonin or receptors in crustaceans to participate in the phagocytosis of foreign pathogens, including several bacteria and WSSV (21).

The C-type lectins (CTLs) were well-characterized among these lectins and highly conserved in crustaceans, which also acted as receptors to bind and agglutinate bacteria or served as opsonins to promote the phagocytosis of bacteria and viruses. Until now, various CTLs have been found to be implicated in phagocytosis in shrimp, crayfish, and crab, including *P*. *monodon, Litopenaeus vannamei, S*. *paramamosain, Fenneropenaeus chinensis*, and *Procambarus clarkii* (Table 1). In *P. monodon, Pm*Lec was found not only to act as a pattern recognition receptor (PRR) to recognize *E. coli* through binding with lipopolysaccharide (LPS) but also to function as an opsonin to enhance hemocyte phagocytosis (25). Since then, several lectins have also been identified in *L*. *vannamei*, which exhibited distinct levels on phagocytic activity against bacteria (27). For instance, the phagocytic rates against *V*. *parahaemolyticus* were decreased to 8.3, 4.5, and 2.5% after silencing the genes of *Lv*CTL5, *Lv*CTLU, and *Lv*LdlrCTL, respectively, in comparison with control groups. It is obvious that the phagocytic activity mediated by *Lv*CTL5 was significantly higher than the other two CTLs. Similarly, both *Pc*Lec3 from *P*. *clarkii* and *Sp*CTL-B from *S*. *paramamosain* could also promote the phagocytic activity of hemocytes against *Vibrio anguillarum* and *V*. *parahemolyticus*, respectively (31, 32). Meanwhile, the expression of phagocytosis-related genes, like *Sp*Myosin, *Sp*Rab5, and *Sp*LAMP [lysosomal-associated membrane protein 1-like protein (LAMP)], could be enhanced by *Sp*CTL-B.

**Table 1:** Phagocytic receptors, ligands, and hemocyte-mediated phagocytosis of pathogens in crustaceans in previous studies.

**Receptors**	**Isoforms**	**Domains**	**Ligands**	**Hemocyte-mediated phagocytosis of pathogens**	**Species**	**References**
C-type lectin	*Pm*Lec	CRD, QPD	LPS	*E*. *coli*	*P*. *monodon*	(25)
	*Lv*Lec	CRD	–	*V*. *alginolyticus*	*L*. *vannamei*	(26)
	*Lv*CTL5	CRD	–	*V*. *parahaemolyticus*	*L*. *vannamei*	(27)
	*Lv*CTLU	CRD	–	*V*. *parahaemolyticus*	*L*. *vannamei*	(28)
	*Lv*LdlrCTL	CRD, LDLR	–	*V*. *parahaemolyticus*	*L*. *vannamei*	(29)
	*Fc*Lec4	QPD	–	*V*. *anguillarum*	*F*. *chinensis*	(30)
	*Sp*CTL-B	CRD	–	*V*. *parahaemolyticus*	*S*. *paramamosain*	(31)
	*Pc*Lec3	CRD, Ig	LPS, PGN, LTA	*V*. *anguillarum*	*P*. *clarkii*	(32)
	*Pc*LT	CRD	Envelope protein VP28, VP19	*V*. *alginolyticus*	*P*. *clarkii*	(33)
L-type lectin	*Mj*LTL1	CRD	LPS, PGN, LTA	*V*. *anguillarum*	*M*. *japonicus*	(34)
Galectins	*Lv*Gal	CRD	–	*V*. *anguillarum*	*L*. *vannamei*	(35)
Scavenger receptors	*Sp*SRB	CD36	–	*V*. *parahaemolyticus and S*. *aureus*	*S*. *paramamosain*	(36)
	*Mj*SRC	MAM, CCP	LPS, LTA, Envelope protein VP19	WSSV, *V*. *anguillarum* and *S*. *aureus*	*M*. *japonicus*	(37, 38)
	*Mj*SRB1	CD36	LPS, LTA	*V*. *anguillarum* and *S*. *aureus*	*M*. *japonicus*	(39)
	*Es*SRB1	CD36	–	*V*. *parahaemolyticus* and *S*. *aureus*	*E*. *sinensis*	(40)
Immunoglobulin-related proteins	*Es*Dscam	Ig, FN	–	*V*. *parahaemolyticus* and *S*. *aureus*	*E*. *sinensis*	(41)
Fibrinogen-related proteins	*Mj*FREP2	FReD	LPS, PGN, envelope protein VP28	*V*. *anguillarum* and *S*. *aureus*	*M*. *japonicus*	(42)

*CRD, carbohydrate recognition domain; QPD, Gln-Pro-Asp domain; LDLR, low-density lipoprotein receptor; Ig, immunoglobulin; SRC, class C scavenger receptor; SRB, class B scavenger receptor; MAM, domain in meprin; CCP, complement control protein domains; WSSV, white spot syndrome virus; Dscam, Down syndrome cell adhesion molecule; FN, fibronectin; FReD, fibrinogen-related domain; LPS, lipopolysaccharide; PGN, peptidoglycan; LTA, lipoteichoic acid; CD36, cluster of differentiation 36; –, not available*.

Apart from C-type lectins, L-type lectins and galectins, another type of lectin, were also identified as important opsonins to promote phagocytosis against bacteria and viruses in crustaceans. Both *Mj*LTL1 and galectin were found to be able to enhance agglutination activity for bacterial clearance and hemocytes phagocytosis against *V*. *anguillarum* in *Marsupenaeus japonicus* and *L*. *vannamei*, respectively (34, 35). However, more types of lectins from various crustacean species and the molecular mechanisms involved in phagocytosis are extremely lacking, which need to be further elucidated for better understanding of lectin-mediated phagocytosis in innate immunity in crustaceans.

### Scavenger Receptors

Scavenger receptors (SRs) are transmembrane proteins that can bind to modified low-density lipoproteins (LDLs), a broad range of polyanionic ligands and cell wall components, which play an essential role in physiological or pathological processes, such as intracellular cargo transport and clearance of pathogen and apoptotic cell (43). SRs occur on the surface of professional phagocytes in both mammalian species and invertebrates, which can be divided into nine heterogeneous classes (A–I) on the basis of their structural diversities (22, 39). SRs generally mediate phagocytosis of non-opsonic pathogens through recognizing PAMPs, including LPS and lipoteichoic acid (LTA). More recently, one of the class B scavenger receptors (SR-Bs), designated as *Sp*SR-B, was found to a be functional phagocytic receptor in the mud crab *S*. *paramamosain*. The *Sp*SR-B recombinant protein could bind onto the surface of *S*. *cerevisiae, V*. *parahaemolyticus, V*. *alginolyticus, A*. *hydrophila, E*. *coli, S*. *aureus*, and β streptococcus. Moreover, *Sp*SR-B-mediated phagocytosis could promote the clearance of *V*. *parahaemolyticus* and *S*. *aureus*. Meanwhile, the expression of phagocytosis-related genes *SpLamp, SpRab5, SpArp*, and *SpMyosin* were significantly downregulated when the *Sp*SR-B gene was knocked down in hemocytes (36). Other studies also proved that class B scavenger receptors (SRBs) could bind to *S*. *aureus* and enhance the phagocytotic rate to facilitate subsequent microbial clearance in *M*. *japonicus* and *E*. *sinensis* (39, 40). In *E*. *sinensis*, the phagocytic rate for *V*. *parahaemolyticus* was decreased from approximately 21 to 15% after silencing of the *EsSR-B1* gene, while the phagocytic rate for *S*. *aureus* was decreased from ~15 to −7%. All the findings indicated that SRB-mediated phagocytosis of *S*. *aureus, V*. *anguillarum*, or *V*. *parahaemolyticus* was very variable with regard to various host species (36, 39).

In addition, the class C scavenger receptors (SR-Cs), similar to that of mammalian class A scavenger receptors (SR-As), specifically recognized LPS of Gram-negative bacteria (22). *Mj*SRC, a class C scavenger receptor characterized from *M*. *japonicus*, was found to be able to mediate phagocytosis of WSSV by binding the viral envelope protein VP19 and then restrict viral replication (37). Meanwhile, *Mj*SRC could also act as a phagocytotic receptor for enhancing phagocytosis and restricting bacterial proliferation through recognizing *V*. *anguillarum* and *S*. *aureus* using its extracellular domains to bind bacterial polysaccharides, such as LPS and LTA (38). Owing to abundant heterogeneous classes of the SRs, it is worthy to further prove whether any other classes of SRs, besides SR-Bs and SR-Cs, are participating in hemocyte phagocytosis in crustaceans against bacterial or viral infection.

### Down Syndrome Cell Adhesion Molecule

Owing to the lack of adaptive immune system for invertebrates, it is commonly accepted that the crustaceans are solely dependent on innate immunity for the organisms against microbial infections. However, a highly variable immunoglobulin-related protein, named as Down syndrome cell adhesion molecule (Dscam), has been identified in both insects and crustaceans (24). As an immunoglobulin super family (IgSF) member, Dscam is composed of a cluster of immunoglobulin (Ig) and fibronectin (FN) domains (37). The first arthropod Dscam, which mostly presents in the neural system, fat body cells, hemocytes, and hemolymph serum in *D*. *melanogaster* (*Dm*Dscam), has been demonstrated as a phagocytic receptor (44). The *Drosophila* Dscam can generate more than 38,000 distinct extracellular domains by mutually exclusive alternative splicing of exons and potentially act as phagocytic receptors and/or opsonin for the recognition of various pathogens. In addition, the phagocytic activity of hemocytes was significantly decreased if the *Dm*Dscam gene expression was silenced in hemocytes (44). Similar to *Dm*Dscam, Dscams identified in *Anopheles gambiae* and *Daphnia pulex* can generate 31,920 and 13,312 alternatives splicing forms, respectively. After silencing of their Dscam gene in *A*. *gambiae* (*AgDscam*), the phagocytic capacity of the Sua5B cells was suppressed against *E*. *coli* and *S*. *aureus*. In crustacean, significantly high diversities of Dscam, generated by a variety of alternative splicing form repertoires with combination of three highly hypervariable Ig domain 2, 3, and 7, have been reported in *L*. *vannamei, P*. *mondon, C*. *quadricarinatus*, and *E*. *sinensis* (41, 45–47). For instance, the variable regions of *Lv*Dscam located in the Ig2, Ig3, and Ig7 domain could potentially encode at least 8,970 unique isoforms, which are speculated to play active roles in response to a spectrum of pathogens (47).

More recently, the *Es*Dscam identified from Chinese mitten crab (*E. sinensis*) could potentially produce over 30,600 isoforms, and certain *Es*Dscam isoforms were found to specifically bind with *S*. *aureus, Bacillus subtilis, A*. *hydrophila*, and *V*. *parahaemolyticus*, respectively, and then could facilitate efficient clearance of the bacteria through phagocytosis. Further investigations revealed that *Es*Dscam contains two forms: soluble Dscam (*Es*-sDscam) and membrane-bound Dscam (*Es*-mDscam). Like an opsonin, coating of *Es*-sDscam onto specific bacteria, such as *S*. *aureus* and *V*. *parahemolyticus*, could enhance the phagocytic ingesting of these bacteria, while such promoting phagocytic function was abolished for the truncated recombinant *Es*-sDscam. With respect to *Es*-mDscam, it served as a phagocytic receptor for *Es*-sDscam through interacting with the same isoform of *Es*-sDscam. In addition, studies have been demonstrated that higher expression level of *Es*Dscam is in parallel to stronger and longer lasting phagocytic capacity of hemocytes in the immune priming *E*. *sinensis* with exposure to *A*. *hydrophila* (48). However, the underlying molecular mechanism of Dscam-mediated phagocytosis as well as its functioning regulation in response to bacterial and viral infections remain insufficient at present. Importantly, whether arthropod Dscam can serve as an antibody analog during the innate immune response against pathogenic infection, especially when we considered about the biological and functional specificity of a pathogen, if there is, remained to be further studied.

### Fibrinogen-Related Proteins

Fibrinogen-related proteins (FREPs, also known as FBNs), containing a highly conserved fibrinogen-related domain (FReD), are one family of the pattern recognition receptors (PRRs) that consist of 53 putative members in *A*. *gambiae*, while only 20 known FREP members in *D*. *melanogaster* (49). The *A. gambiae* FREPs family has been proven to play a crucial role in mosquito against bacteria and malaria parasites, in which FREPs could bind with *E*. *coli, Pseudomonas veronii*, and *Beauveria bassiana*. FBN9 can form dimers for binding to the bacterial surfaces with different affinities (23). FREPs have also been reported to play an important role in the innate immunity of crustaceans. For instance, fibrinogen-related proteins were identified from *M*. *japonicus* (*Mj*FREP1 and *Mj*FREP2) (19, 42), which exhibited different expression levels in different tissues. Both *Mj*FREP1 and *Mj*FREP2 could bind to *V*. *anguillarum* and *S*. *aureus* through interaction with LPS and peptidoglycan (PGN). Furthermore, *Mj*FREP2 could recognize the invading bacteria and facilitate bacterial clearance by promoting the phagocytosis of hemocytes. However, the molecular mechanism underlying hemocyte phagocytosis mediated by FREPs still needs further investigations, in consideration to their key roles in innate immunity.

## Signaling Cascades Involved in Phagosome Formation and Maturation

After coating with opsonin and recognition of the pathogens by related phagocytic receptors, a series of signaling events for initiating phagocytosis will be thereby triggered, and will result in remodeling of the actin cytoskeleton to produce membrane pseudopods for internalizing the pathogen to form phagosome (50). Then, phagosomes gradually fuse with early and late endosomes and eventually fuse with lysosome to generate phagolysosome. Importantly, small GTPases of the Rab family represent a key cluster of molecules that closely related to phagosome formation and maturation by modulating the remodeling of cytoskeleton. Although no direct evidence about phagosome formation and maturation for engulfing and ingesting microorganism was demonstrated in crustacean, to our best knowledge, several important components, mainly small GTPases members such as Rab6, Rab7, RhoA, and Ran, have been reported in some species of shrimp for their involvement in the signaling cascades of the highly conserved processes (51–54). Most of them have also been proven previously to play essential roles in regulating the actin polymerization and dynamic remodeling to form phagosome in vertebrates (51).

**Figure 1:**
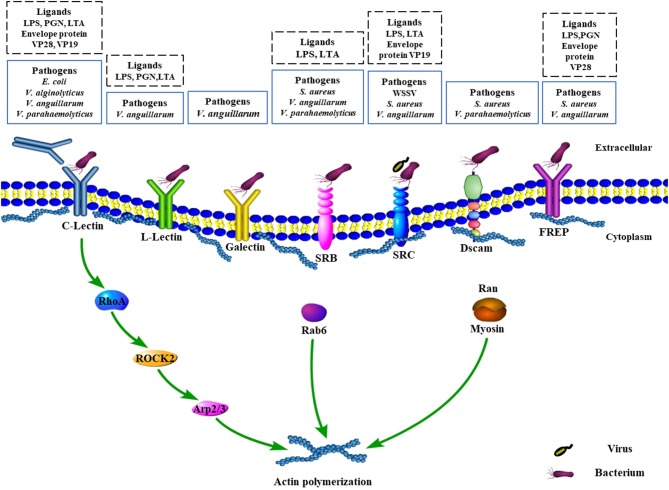
Schematic representation of recognition of pathogens by surface receptors and signaling pathways involved in phagocytosis in crustaceans. C-Lectin, L-Lectin, galectin, SRB, SRC, Dscam, and FREP are selected and modified from previous publications. For more details of all the molecules, please refer to the related references. C-Lectin serves as opsonin or receptor to recognize the invading bacteria and regulate the expression of downstream factor RhoA, which further stimulates the activity of the ROCK2 and Arp2/3 to participate in actin polymerization (51). In addition, Rab6 participates in actin polymerization. Meanwhile, Ran interacts with myosin to participate in actin polymerization. ROCK2, serine/threonine kinase Rho kinase 2; SRC, class C scavenger receptor; SRB, class B scavenger receptor; Dscam, Down syndrome cell adhesion molecule; FREP, fibrinogen-related proteins.

Rab7 is a well-known key protein that localizes on the membrane of late endosome and plays a critical role in the phagosome maturation, which has also been found to promote the phagocytosis against *S*. *aureus* and *V*. *parahaemolyticus* in hemocytes of *E*. *sinensis* (55). However, the role of Rab7, as well as the conversion of Rab5 to Rab7, is still poorly understood in crustaceans. Another Rab family member termed as *Pj*Rab6, was found to directly interact with β-actin, tropomyosin, and the WSSV envelope protein VP466 for generating a complex to regulate hemocytic phagocytosis in *P*. *japonicus* (56). Interestingly, a further study found that viral envelope protein VP466 was employed by the host cell to increase the GTPase activity of PjRab6 to induce rearrangements of the actin cytoskeleton for the formation of actin stress fibers and subsequently promote the phagocytosis against WSSV (57). All findings indicated that phagocytosis is a complicated process, in particular, phagosome formation and maturation, which not only require host factors but also is associated with the utilization of pathogens components. Moreover, as an important member in phagosome formation and maturation, it is not surprising that *Pj*Rab6 could also increase the hemocytic phagocytosis against bacteria, such as *V*. *parahaemolyticus*, although the regulatory molecular events involved in the phagocytosis of bacteria are still unclear [Figure 1; (54)].

Besides, as one of the Ras GTPase superfamily members, *Mj*RhoA has also been identified in *M*. *japonicus* to be able to induce the expression level of phagocytosis-related genes ROCK2 and Arp2/3 as well as to promote the phagocytosis rate against *V*. *anguillarum* in hemocytes (51). Both ROCK2 and Arp2/3 were reported to be implicated in the integrin-mediated phagocytosis pathway (51). Previously, a C-type lectin receptor, *Fc*Lec4, was found to be able to facilitate phagocytosis and bacterial clearance through binding to its receptor β-integrin in *F*. *chinensis* (30). Further study showed that *Mj*RhoA participated in hemocytes phagocytosis against *V*. *anguillarum* infection via β-integrin-dependent signaling pathway, which was the downstream factor for β-integrin (51). Ran GTPases, another family of the small G protein superfamily, has also been found to be involved in phagosome formation and maturation in crustacean hemocytes. For example, *Pj*Ran, a Ran GTPase identified from *P*. *japonicus*, has been proven to modulate hemocytic phagocytosis against WSSV via interaction with the cytoskeleton protein myosin (58). Given the importance and contribution to modulate phagocytosis, researches on exploring more members of the small GTPases family are necessary for understanding the molecular mechanism underlying phagosome formation and maturation against pathogens. Beyond that, the development of phagolysosome also needs to recruit more host components, such as the vacuolar ATPase and NADPH oxidase complex, while various organelles including mitochondria, the endoplasmic reticulum, and Golgi-derived vesicles are also required to make contributions to this complicated physiological process (50). However, extremely poor knowledge is available about that due to limited studies conducted in this field in crustaceans.

## Other Factors Involved in Phagocytosis

Although lots of molecular mechanisms in phagosome formation and maturation have not been well-investigated in crustacean, however, increasing evidence showed that some other factors could also modulate phagocytosis against bacteria. Among them, antimicrobial peptides (AMPs) have been reported as one of the most important components to participate in phagocytosis in crustaceans.

AMPs have been well-characterized as important effectors in the innate immune system in both insect and crustacean, which are mainly expressed in hemocytes and then released into the hemolymph for defensing against a broad spectrum of microorganisms (59). Until now, several types of AMPs, such as penaeidins, antilipopolysaccharide factors, and crustins, have been discovered in crustaceans. Interestingly, in addition to their antimicrobial activity, some AMPs also seem to participate in the phagocytosis of pathogens during microbial infection in shrimp.

Penaeidins are one family of antimicrobial peptides that consist of proline-rich N-terminals and a C-terminal cysteine-rich region (60). To date, ~40 penaeidins have been identified in shrimp, which belong to five types based on their similarity of amino acid sequences. Recently, a penaeidin with an additional serine-rich region (*Mj*Pen-II) from *M*. *japonicus* showed not only antimicrobial but also phagocytic activity (61). The rate of phagocytosis was significantly decreased after the *Mj*Pen-II gene was silenced, while compensation with injection of *Mj*Pen-II recombinant protein *in vivo* could increase the phagocytic activity. Further study showed that *Mj*Pen-II could eliminate bacteria through directly inhibiting bacterial growth as well as promoting phagocytosis.

Crustins are another type of antibacterial peptides that contain a single whey acidic protein domain at the C-terminus. In shrimp, two isoforms of crustins, including *Mj*Cru I-1 and crustin-like peptide, have been characterized to be associated with phagocytosis, which were increased in hemocytes after challenge with bacteria (62). Meanwhile, both of them exhibited binding activity to bacteria followed by the increased phagocytosis of hemocyte. In addition, the phagocytic rate of WSSV was significantly decreased when the crustin-like gene was knocked down in shrimp hemocytes, whereas the phagocytic rate of *V*. *alginolyticus* was increased in hemocytes, exhibiting different biological effect against microorganisms (62). However, the underlying regulatory molecular mechanism of AMP-mediated phagocytosis is still extremely limited, which is worthy of further investigations.

Except AMPs, some other molecules have also been reported to regulate phagocytosis of invading pathogens in crustaceans. For instance, the recombinant phagocytosis-activating protein (PAP) was shown to significantly promote the phagocytic activity of hemocytes in *P*. *monodon* (63). Two neuroendocrine factors, crustacean hyperglycemic hormone (CHH) and dopamine, were also found to participate in phagocytosis in *L*. *vannamei* hemocytes. CHH exhibited regulating capacity on phagocytosis through activating nuclear factor kappa B (NF-κB) signaling pathway family members and phagocytosis-related proteins, while dopamine acted as a phagocytosis-related factor to inhibit phagocytosis (64, 65). Except for proteins, some microRNAs (miRNAs) have also been found to play critical roles in regulating phagocytosis process in crustaceans. In hemocytes of *M*. *japonicus*, miR-1 has been found to negatively regulate the phagocytic activity through interaction with the 3′ untranslated region (UTR) of clathrin heavy chain 1 (CLTC1) gene (66). Moreover, miR-12 and miR-965 were also found to be able to enhance the phagocytosis of WSSV via direct targeting phosphatase and tensin homolog (PTEN) and viral gene wsv240, respectively (67, 68), while another miR-100 could promote the antibacterial and antiviral immune response but through regulating the total hemocyte count and phagocytosis in *M*. *japonicus* (69). In addition, the shrimp-specific miR-S5 acted as a regulator on hemocyte phagocytic progress via negatively regulating myosin expression (70). Taken together, miRNAs act as regulator involved in antibacterial or antiviral immune responses through direct targeting viral genes or cytoskeleton-related genes accordingly. However, the specific regulatory mechanism of miRNA involved in phagocytosis against pathogens needs to be further studied.

## Conclusion and Future Perspective

Although several cellular surface receptors/opsonin and intracellular regulator for mediating phagocytosis against bacteria and viruses have been reported in hemocytes from multiple species of crustaceans, limited studies make it extremely unclear until now about the molecular mechanism of recognition of pathogens and the downstream signaling events, in particular those in relation to phagosome formation and maturation, as well as microbe destruction in crustaceans. Future research will be focused on the following fundamental questions in relation to the above concerns. For instance, besides the three described types of hemocytes involved in the phagocytosis in crustaceans, are there any other phagocytic cells types present in crustaceans and additional surface receptors involved in pathogen recognition? What are the regulatory mechanisms for various phagocytic receptors located on hemocytes to cooperate with each other during phagocytosis of different microorganisms? More importantly, are the processes of phagosome formation and maturation in crustaceans similar to that in vertebrates? Last but not least, how does the cooperation occur among various intracellular regulators to mediate phagocytosis, and are there other novel signaling pathways involved in the regulation on hemocyte-mediated phagocytosis? Successfully addressing these important questions will pave avenues to deeper understanding of phagocytosis against microbial infection in crustaceans, in which the fundamental mechanism of phagocytosis in crustacean will benefit the establishment of more efficient control strategies against disease in crustacean farming.

## Author Contributions

SL and S-CZ wrote the manuscript. Y-LL contributed to suggestion and discussion. JL and H-PL designed and finally polished the manuscript.

### Conflict of Interest

The authors declare that the research was conducted in the absence of any commercial or financial relationships that could be construed as a potential conflict of interest.
